# Production and Application of Biosurfactant Produced by *Bacillus licheniformis* Ali5 in Enhanced Oil Recovery and Motor Oil Removal from Contaminated Sand

**DOI:** 10.3390/molecules24244448

**Published:** 2019-12-04

**Authors:** Nawazish Ali, Fenghuan Wang, Baocai Xu, Bushra Safdar, Asad Ullah, Muhammad Naveed, Ce Wang, Muhammad Tayyab Rashid

**Affiliations:** 1 Beijing Advanced Innovation Center for Food Nutrition and Human Health, Beijing Technology & Business University (BTBU), Beijing 100048, China; Alibtbu@gmail.com (N.A.); bushrasafdar1@gmail.com (B.S.); asadbahi2016@gmail.com (A.U.); nnourang@yahoo.com (M.N.); wangce@btbu.edu.cn (C.W.); 2 School of Light Industry, Beijing Technology & Business University (BTBU), Beijing 100048, China; 3 Food and Marine Resources Research Center, PCSIR Laboratories Complex, Shahrah-e-Dr. Salim-uz-Zaman Siddiqui, Karachi 75280, Pakistan; 4 School of Food and Biological Engineering, Jiangsu University, Zhenjiang 21050, China; trashid208@gmail.com

**Keywords:** lipopeptide biosurfactant, surface tension, interfacial tension, wettability alteration, sand packed column

## Abstract

The present study describes the production of biosurfactant from isolate *B. licheniformis* Ali5. Seven different, previously-reported minimal media were screened for biosurfactant production, and two selected media were further optimized for carbon source. Further, various fermentation conditions such as (pH 2–12, temperature 20–50 °C, agitation speed 100–300 rpm, NaCl (0–30 g·L^−1^) were investigated. The partially purified biosurfactant was characterized by Fourier transform infrared spectroscopy (FTIR) and matrix-assisted laser desorption/ionization time-of-flight mass spectroscopy (MALDI-TOF MS) and found a lipopeptide mixture, similar to lichenysin-A. Biosurfactant reduced surface tension from 72.0 to 26.21 ± 0.3 and interfacial tension by 0.26 ± 0.1 mN·m^−1^ respectively, biosurfactant yield under optimized conditions was 1 g·L^−1^, with critical micelle concentration (CMC) of 21 mg·L^−1^ with high emulsification activity of (E24) 66.4 ± 1.4% against crude oil. Biosurfactant was found to be stable over extreme conditions. It also altered the wettability of hydrophobic surface by changing the contact angle from 49.76° to 16.97°. Biosurfactant efficiently removed (70-79%) motor oil from sand, with an efficiency of more than 2 fold as compared without biosurfactant (36–38%). It gave 32% additional oil recovery over residual oil saturation upon application to a sand-packed column. These results are indicative of potential application of biosurfactant in wettability alteration and ex-situ microbial enhanced oil recovery.

## 1. Introduction

Crude petroleum demand increases day by day as a result of global urbanization and industrialization. Previous studies demonstrated that around 60%–70% of crude oil is still trapped in oil reservoirs after conventional oil-recovery methods [1]. To secure the demand, more efficient and economical oil extraction from the depleting oil resources needs some enhanced oil-recovery (EOR) technologies, which include chemical, microbial and miscible gas injections into oil wells [2,3,4] Among different EOR methods, chemical surfactants and polymers are the most utilized compounds in enhanced oil recovery. However, these chemical-enhanced oil-recovery methods are environmentally hazardous and leave undesirable residues [5]. The microbial biosurfactants are worthy alternatives to the conventional chemical EOR methods. Microbial surfactants or biosurfactants can be produced by different species of bacteria and unicellular fungi and yeast. These biosurfactants have surface active molecules which reduce the interfacial tension between an aqueous phase and hydrophobic molecules because of their amphipathic nature. In addition, biosurfactants increase bioavailability and solubilization of organic hydrophobic compounds [6]. The recent advancement in microbial-enhanced oil recovery (MEOR) technology and usage of cheaper substrate and economical bio-manufacturing applications not only improves the yield but has also improved the economical production and utilization approaches for microbial-enhanced oil recovery [7,8]. A few reports have authenticated the application and success of the ex situ enhanced oil recovery method at a laboratory scale, and reported an additional 10%–85% oil recovery using core floods or sand pack columns [9,10]. The lipopeptide surfactins and lichenysin produced by *Bacillus subtilis* and *Bacillus licheniformis* are among the most studied biosurfactants. These biosurfactants are known for lowering surface and interfacial tensions along with other properties and they are capable of mobilizing entrapped oil and hence are good candidates for MEOR application [11,12]. 

Biosurfactants have amphiphilic moieties produced extracellularly by a wide range of bacteria, fungi and yeast. Biosurfactants are chemicaly classified into into lipopeptides, neutral lipids, glycolipids, phospholipids, polymeric and particulate compounds [13,14]. Biosurfactants act between fluids with different polarities such as oil and water, aiding access to hydrophobic substrates by increasing the area of contact of insoluble compounds as well as improved bioavailability and mobility, leading to the biodegradation of these substrates. These properties enable biosurfactants to lower surface and interfacial tensions and also form microemulsions. These features make hydrocarbons solubilized in water [15]. Spillage of hydrocarbons is another major problem that contributes in pollution of soil and marine environments. The removal of these hydrocarbon pollutants is very difficult because of their insoluble nature. Biosurfactants again efficiently participate in biodegradation, resulting in removal of these organic and inorganic pollutants [16]. 

The mechanical workshops contribute thousands millions gallons of used engine oils annually and are released without any treatment, it is reported that a liter of waste engine oil can pollute one million gallon of fresh water. Hence, the remediation of waste engine oil is mandatory for environmental safety [17]. Biosurfactants’ addition can improve bioremediation of pollutants with indigenous cultures due to incredible features like emulsification and de-emulsification, dispersion foaming and wetting [18]. Synthetic surfactants have been utilized for enhanced soil washing, including anionic, nonionic, cationic and mixed surfactants, some of these surfactants have proved their worth in washing capabilities for removal of organic and inorganic hydrocarbons from contaminated underground water and soils [19]. Synthetic surfactants like TritonX-100 and Tween 80 are able to enhance the concentration of non-polar compounds in the aqueous phase [20]. However, the synthetic surfactants exert toxic hazards to the environment and human health so biosurfactants are suitable alternative [9].

Thus, the aim of the present study includes: the screening and isolation of biosurfactant producing bacteria from soil, improvement of biosurfactant yield through selection of appropriate media and better carbohydrate source, characterization of the produced biosurfactant and access to its stability in harsh environmental conditions. Further, the biosurfactant’s applicability was assessed for environmental applications like enhanced oil recovery in sand-packed column along with its capacity to remove hydrocarbons from contaminated sand.

## 2. Results and Discussions

### 2.1. Screening and Identification of the Bacterial Isolate

In the present study, three morphologically distinct microbial colonies were isolated, and in particular the isolated strain named initially “10^−2^ w” was recorded with the highest surface tension reduction (26.37 mN·m^−1^), hemolytic activity (positive), oil spreading (65 mm), positive result in drop collapse and emulsification index (E_24_) with crude oil as 66%. Thus, it was selected for further studies. 

The selected bacterial strain after screening parameters was identified following the technical route map: Bacterial genomic DNA extraction → 16S rRNA bacteria conserved sequence polymerase chain reaction (PCR) amplification → 3730 sequencing. Genomic DNA from selected colonies of 10^−2^ W and 10^−3^ W were extracted, universal primers 27f: AGAGTTTGATCMTGGCTCAG and 1492R: TACGGYTACCTTGTTACGACTT were used for 16S rRNA sequencing. The specific sequence obtained was compared with the database of known 16S rRNA, using BLAST (basic local alignment search tool) the strain was identified as *Bacillus licheniformis* and named *B. licheniformis* Ali5. The 16S rRNA sequence of the strain *B. licheniformis* Ali5 was submitted to the Gen-Bank database with accession number MN629226.

**Figure 1:**
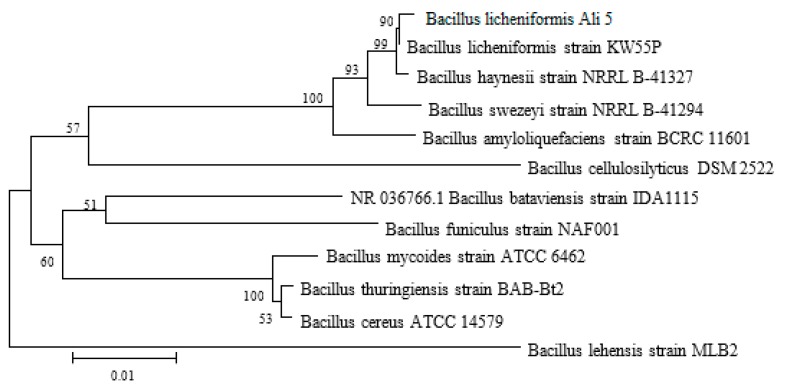
Phylogenetic tree of the strain through neighbor-joining methods using MEGA version 6.0. It represents how Ali5 belongs to *B. licheniformis.*

The phylogenetic tree of the strain is shown in (Figure 1).

### 2.2. Media and Carbon Source Optimization

Microbial-enhanced oil recovery is an old technique that commenced as far back as 1926, and was formerly called “MIOR” (microbial increased oil recovery) where both microbial products and microorganisms are used for oil recovery from depleted oil wells and reservoirs. To ensure adequate energy supply in the future, MEOR is very useful [21]. Several biosurfactants reported for playing a crucial role in enhancing oil recovery by stimulating dissolution, solubilization and emulsification of hydrocarbons [22]. Lipopeptides are among several other biosurfactants that have found application in microbial enhanced oil recovery. Previously, some endospores were used for microbial enhanced oil recovery collected from soil having a hydrocarbon history. Thus, seven different minimal media were used in the present study to investigate the production of biosurfactant, pH variations, surface and interfacial tension and viscosity of the biosurfactant at different time intervals (24–72 h). The growth and production profile in seven minimal media are shown in Table 1. It was observed that media M2, M6 and M7 have better growth, while very little growth was observed in media M3 and M4. The possible reason for this may be the acidic pH after 24 h of growth while the rest of the mediums have neutral to alkaline pH. This strain showed better growth in neutral to alkaline pH, as previously reported by Joshi et al. [23]. M2 and M7 both had the lowest surface tension and interfacial tension of 26.37–27.33 mN·m^−1^ and 0.78–2.60 mN·m^−1^, respectively

Based on these results, media M2 and M7 were selected further for better carbohydrate source investigation. Both M2 and M7 media were selected for screening of carbon source (glucose, sucrose and starch). The results obtained indicating better growth, lower surface tension (ST) and interfacial tension (IFT), high emulsification index (E_24_) and yield in M7 with all carbon sources. It was observed that all media containing glucose, sucrose and starch as carbon source reduced ST from 72 to ˂29.6 mN·m^−1^ and similar trend was observed in M7 as surface tension dropped from 73 to 26.37 and interfacial tension from 48.66 to 0.26 mN·m^−1^ within 72 h, highest emulsification 54.4% (Table 2). No media indicated considerable viscosity variations during 72 h of growth. Hence, it may be conluded that the strain *B. licheniformis* Ali5 did not produce biopolymer but produced only biosurfactant. The viscosity of some biopolymers was studied and found to be around 43–21,535 cP [23]. These results are in accordance with some of the previously reported bacillus species [24,25,26]. It is concluded from the results that the isolated strain *B. licheniformis* Ali5 gave better growth, ST, IFT, E_24_ and yield with glucose as carbon source. Hence, M7 with glucose as a carbon source was used for further studies. The yield of biosurfactant at different time intervals and with different hydrocarbon sources was studied and the biosurfactant was extracted by combination of acid-precipitation and methanol extraction, and then freeze-dried. The obtained yield of partially purified biosurfactant was 1.10 ± 0.2 g·L^−1.^

### 2.3. Effect of Environmental Conditions on Growth and Stability

**Figure 2:**
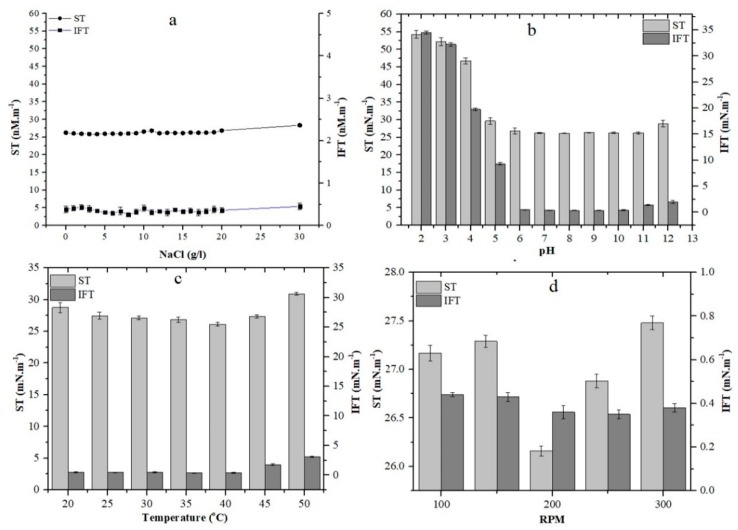
Effect of salinity (**a**) pH (**b**) temperature (**c**) agitation speed rotation per min (RPM) (**d**) on growth (Surface Tension and Interfacial Tension) of biosurfactant produced by *B. licheniformis* Ali5. The error bars represent standard deviations from three independent experiments. SD (*n* = 3).

The variations in NaCl concentrations, pH, temperature and agitation speed on growth were determined by measurement of surface and interfacial tension, as shown in Figure 2a–d. The effect of pH on ST and IFT was greatly affected by changes in pH below 4 while the pH around 4–12 leads to only a slight change in ST and IFT. The better growth was observed towards the neutral and alkalined environment. It was also observed that the agitation speed from (100–300 rpm) and NaCl up to (30 g/L) had no significant effect on surface and interfacial tension. No significant change in ST and IFT were observed at a temperature around 20–40 °C and a slight increase in ST and IFT were seen from 40 to 50 °C. 

**Figure 3:**
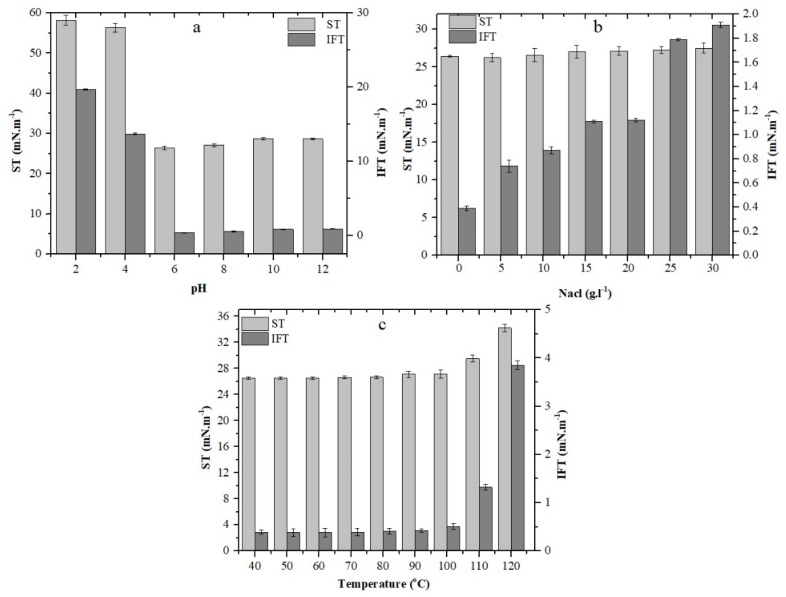
Effect of pH (**a**), salinity (**b**), temperature (**c**) on the stability (surface tension and interfacial tension) of biosurfactant produced by *B. licheniformis* Ali5. The error bars represent standard deviations from three independent experiments. SD (*n* = 3).

Stability in harsh environmental conditions is considered mandatory for all chemicals to find their application in additional oil recovery (AOR). Thus, the biosurfactant was exposed to higher temperatures, pH and salinity as shown in Figure 3. The studied range of temperatures were 40–110 °C, 121 °C (15Psi for 30 min) and the biosurfactant was found to be stable up to 100 °C, while a negligible change in ST and IFT was observed at 110 °C and 121 °C. Banat et al. [27] reported that heat treatment even after autoclaving cannot change the properties of some biosurfactants. The ST and IFT values slightly change when the pH increased from 6–12 but at pH below 4 the ST and IFT values significantly increased due to precipitation (Figure 3a). These results indicate that increased pH had a positive effect on surface, interfacial activity and stability of biosurfactant [28].The surface activity of the biosurfactant was accessed under different salinity concentrations and the biosurfactant performed well in a salinity test up to 30 g·L^−1^ (Figure 3b). The surface activity remained higher even at the highest salt concentration of (30 % *w/v*), by which it was not effected, and it exhibited high stability. Some reports have confirmed similarly good stabilities of biosurfactants produced by *Bacillus* and *Pseudomonas aeruginosa* strains [18,29,30]. These properties of biosurfactants make them an exceptional candidate for application in microbial enhanced oil recovery, since biosurfactants are excellent alternatives to chemical surfactants that are toxic, non-degradable and less activated at 4%–5% salinity, as discussed by Bognolo et al. [31].

### 2.4. Determination of Emulsification Index E_24_

The emulsification activity of biosurfactant containing supernatant of M7 media having glucose as a carbon source was appreciable. Emulsified hydrocarbons like kerosene, hexadecane, tridecane, tetradecane, diesel, crude oil, pristane and heptane were in the range of 50%–64%. As shown in Table 3 the biosurfactant-produced emulsions were more stable than emulsions produced by SDS or Triton X. Biosurfactant supernatant exhibited higher E_24_ values of 66.4%, 57.6% and 56.5% against crude oil, kerosene and tetradecane, respectively. de Faria et al. [25] reported that the biosurfactant from bacillus species emulsified hydrocarbons, including hexadecane, kerosene, diesel, petrol and benzene in the range of 30%–80%. The obtained results were similar to those obtained by Nitschke et al. [12]. A higher emulsification property (66.4%) was observed with Xinjiang crude oil, which is an indicator of possible biosurfactant usage in enhanced oil recovery and environmental application.

### 2.5. Critical Micelle Concentration (CMC) Determination

**Figure 4:**
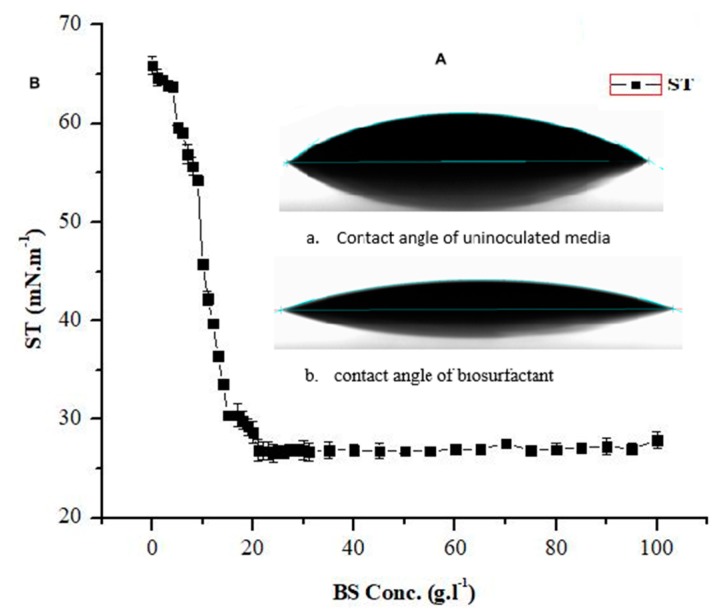
A (**a**) Contact angle of un-inoculated media (49.76 ± 1.4°) (**b**) biosurfactant from *B. licheniformis* Ali5 (cell-free supernatant) (B) critical micelle concentration (CMC) of the biosurfactant produced by strain Ali5. The error bars represent standard deviations from three independent experiments. SD (*n* = 3).

As shown in (Figure 4b), the freeze-dried biosurfactant dissolved in Tris-HCL buffer solution pH 8 was used to determine the CMC value and was found to be 21 mg·L^−1^ with reduced surface tension to 26.42 mN·m^−1^. Almost constant surface tension was observed up to 1 g·L^−1^ concentration of biosurfactant. These results are in agreement with Joshi et al. [28] and much higher than Al-Sulaimani et al. [3], and relatively lower than some other biosurfactants produced by some other *Bacillus* species [32,33,34].

### 2.6. Surface Wettability Alteration

Surface wettability alteration has been reported as a significant mechanism for the EOR process. Wettability alteration is correlated with interfacial tension that can change water-wet to oil-wet biosurfactant surfaces, and vice versa [26]. Therefore, biosurfactant (cell-free supernatant) produced from the *B. licheniformis* Ali5 was studied for any effect on the hydrophobic surface of a cover slip. The results obtained for contact angle measurements are shown in (Figure 4a,b) the contact angle was reduced from 49.76 ± 1.4° of un-inoculated media to 16.97 ± 0.84° in cell-free supernatant. Joshi et al. [23] obtained results for un-inoculated media and cell-free supernatant from 55.67 ± 1.6° to 19.54 ± 0.7° respectively. Al-Wahaibi et al. [26] found the contact angle alteration from 70.6 ± 0.3° to 27.4 ± 1.03° using biosurfactant from *B. subtilis* B30. In the current study, the biosurfactant altered wettability to make it more water-wet and this is considered a suitable property in improving the field-scale enhanced oil recovery applications. 

### 2.7. Partial Structural Identification Using Fourier Transform Infrared Spectroscopy (FTIR) 

**Figure 5:**
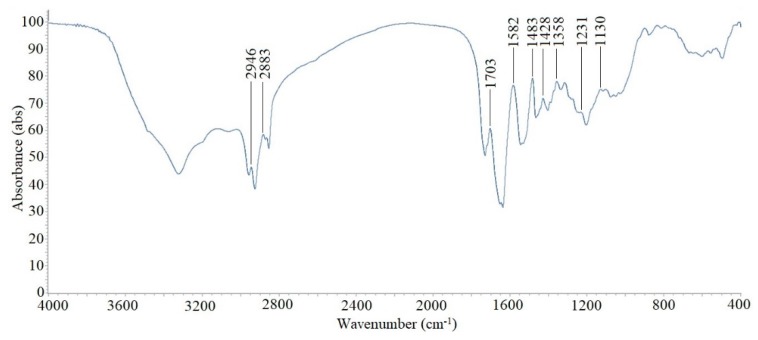
Fourier transform infrared spectroscopy (FTIR) spectrum of the biosurfactant produced by *B. licheniformis* Ali5.

The FTIR spectrum (Figure 5) of the partially purified biosurfactant of *Bacillus licheniformis* Ali5 showed a similar pattern to surfactin (Sigma chemicals, USA) and peaks obtained indicate that the biosurfactant is a lipopeptide, as observed by Joshi et al. [28]. Band characteristics of the peptides were at 3323 cm^−1^ (NH stretching mode), and at 1629 cm^−1^ (stretching mode of CO-N bond); while the bands at 2957–2926, 2854, 1403, 1377 cm^−1^ reflected the aliphatic chains (-CH3, -CH2-) of the fractions, as described in Joshi et al. [28]. It is concluded from the results that the biosurfactant is a cyclic lipopeptide, the infrared spectrum produced is quite similar to bacilli produced Surfactin and Lichenysin as reported previously [3,23].

### 2.8. MALDI TOF-MS of Produced Biosurfactant

**Figure 6:**
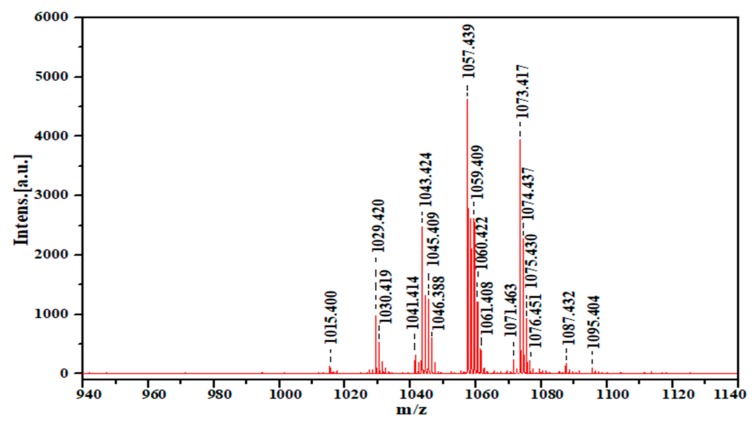
The matrix-assisted laser desorption/ionization time-of-flight (MALDI TOF) spectrum of the biosurfactant produced by *B. licheniformis* Ali5.

Matrix-assisted laser desorption/ionization Time-of-flight mass spectrometry (MALDI-TOF) of the purified biosurfactant (acid precipitation/methanol extract) of *B. licheniformis* Ali5 strain is shown in (Figure 6). The positive mode operated MALDI-TOF demonstrated mainly the sodium adducts peaks of the compound. A total of 18 peaks were observed having mass to charge ratio within the range from 1015.400–1095.404. [M + Na]^+^ ions with mass to charge ratio 1015.400, 1029.420, 1030.419, 1043.424, that correspond to the heptapeptide moiety a characteristic of surfactants linked to C13, C14 and C15 hydroxy fatty acid chains. [M + K]^+s^ with mass to charge ratio of 1087.432. The results of the analysis of *B. licheniformis* Ali5 produced biosurfactant indicated that the main fractions contain lipids and peptide moieties, which have similarities to lichenysin-A and their homologs. Previous studies [23,35,36] authenticated the biosurfactant from *B. licheniformis* as cyclic heptalipopeptide, having a small peptide chain (Gln, Leu, Val, Asp, lle) that linked to 3-3-hydroxylated tri, tetra and pentadeconoic acids.

### 2.9. Biosurfactant Application in Hydrocarbon Removal from Contaminated Sand

**Figure 7:**
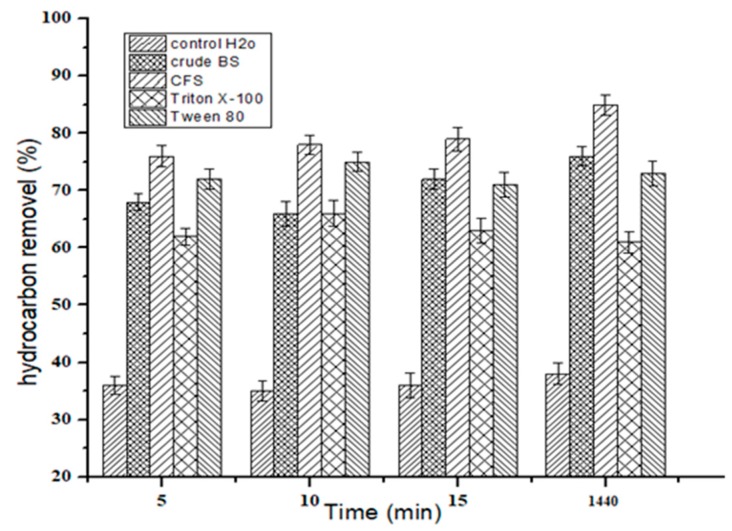
Assessment of crude biosurfactant, CFS = cell-free supernatant, Triton X-100 and Tween 80 on removal of 10% (*v/w*) used motor oil from sand. The error bars represent standard deviations from three independent experiments. SD (*n* = 3).

The degradation and removal of petroleum hydrocarbons bound to the soil is difficult. Surfactants can increase the displacement of hydrocarbons from the soil by making them more water-soluble, lowering their ST and IFT, and by emulsifying hydrocarbons [27]. For the investigation of the hydrocarbon removal from contaminated sand, the 72 h-old cell-free supernatant of M7 glucose-based media and solution of crude biosurfactant was used while the synthetic surfactants Triton X-100, SDS, Tween 80 and Tween 20 at their CMC levels and water as control were used to perform the potential hydrocarbon removal from the sand. Biosurfactant (cell-free supernatant) recovered 79% (3.9 g) of used motor oil from sand and crude BS solution 70% (3.5 g), while Triton X-100 63% (3.15 g), Tween 80 recovered 72% (3.6 g) respectively. Water after 24 h recovered 36-38% (1.8 g) of motor oil from contaminated sand. The biosurfactant successfully recovered more than 2 fold extra oil from sand in comparision with the control (Figure 7). Cell-fee supernatant (CFS) showed promising abilities of hydrocarbon removal from contaminated sand. Biosurfactants are preferred over synthetic surfactants since synthetic surfactants are toxic and have low biodegradability and are harmful to the ecosystem [37]. The cell-free supernatant had slightly higher removal ability then crude BS. This indicates that the cell-free supernatant can be directly used, which can reduce the purification expenses of biosurfactants [38]. Similarly, it was reported that the CFS was as effective as purified biosurfactant; BS from *P. aeruginosa* removed 80% hydrocarbons from the sand [39]. Therefore, it may be suggested that the isolated bacterial strain of *Bacillus licheniformis* Ali5 is a potential candidate for metabolic enhanced oil recovery, bioremediation and in oil industries as it implies a lower cost of biological surfactant production and lower purity specifications.

### 2.10. Enhanced Oil Recovery Using Sand-Packed Column Test

To evaluate the potential ability of biosurfactant in ex-situ microbial enhanced oil recovery, biosurfactant was flooded into a sand-packed column. Sand packed in the column is uniform, but it varies in structure and porousness, exhibiting differences in pore volumes from 59–62 mL, original oil in place (OOIP) 45–48 mL, Sorwf 23.6–26.4 mL and Sorbf) from 7.5–8.5 mL in a set of three columns used. Biosurfactant flooding was engaged for additional oil recovery as the water flooding was incapable of recovering the entrapped oil. The water flooding resulted in 7.1 ± 1.4 additional oil recovery. Additional oil recovery using biosurfactant (cell-free supernatant) by *B. licheniformis* Ali5 resulted in (4.5 fold increased) 32.10 ± 0.4% as shown in (Table 4). It was previously reported by Jha et al. [10] that biosurfactants from *B. subtilis* R1 recovered 33 ± 1.2% additional oil using a sand-packed column. Darvishi et al. [30] reported 27% additional oil recovery with crude biosurfactant and cell-free supernatant using core flood experiment. Wahaibi et al. [26] reported that crude biosurfactant from *B. subtilis* enhanced light oil recovery by 17%–26% and 32% of additional heavy oil recovery. These results are in agreement with Suthar et al. [40] Gudina et al. [41] and Sun et al. [42] who studied potential ability of biosurfactants using a sand-packed column and reported additional oil recovery below 50%. The biosurfactant produced can desorb entrapped oil from the rocks as it lowers the interfacial tension and makes a stable emulsion, leading to mobilization of crude oil. It brings oil in water phase as the surfactant and it exerts mobility of oil like a polymer. Hence, the mechanism of bioemulsifier is envisaged as surfactant-polymer flooding. These results suggest that microbial metabolites having a combination of surface and interfacial activity along with significant emulsification index (E24) have promising AOR ability. It was also found that the sand-packed column construction was inexpensive, easy and effective. Thus, the sand-packed column performed as a suitable bench-scale method to test the potential ability of biosurfactant in additional oil recovery. 

## 3. Materials and Methods

### 3.1. Chemicals/Reagents and Isolation of Biosurfactant-Producing Bacteria

Soil samples with a history of hydrocarbon waste were collected from different locations of the Haidian district of Beijing, PR China. The samples were collected in sterile polyethylene bags, labeled and taken to the laboratory for further study. At regular time intervals, sample moisture was maintained with 0.85% saline water and stored at room temperature [43]. Samples were serially diluted up to 10^−6^ dilutions and plated into mineral salt agar medium (MSM) (1.8 g K_2_HPO_4_, 4.0 NH4Cl, 0.2 g MgSO4.7H2O, 0.1 g NaCl, 0.01 g FeSO4.7H2O, 15 g agar in 1 L of distilled water, 1.0% (*v/v*) Xinjiang crude oil was used as sole carbon source), incubated at 37 °C for 16 hours. Morphologically different colonies were re-streaked into nutrient agar plates to obtain a pure culture. Colonies were then tested for biosurfactant activity by using hemolytic activity, oil displacement test, emulsification activity and direct surface tension measurement (ST), isolated bacterial colonized are stored in a refrigerator at 4 °C as stock culture. All chemicals and hydrocarbons (hexadecane heptane, tridecane, tetradecane and pristane) were purchased from (Sigma-Aldrich, Co USA). Light crude oil was provided by Xinjiang oilfield, China. Kerosene and diesel were purchased from local markets.

### 3.2. Screening Assays for Biosurfactant Producers

#### 3.2.1. Oil-Spreading Method

For the oil displacement test, a 90 × 10 mm petri dish was filled with 50 mL distilled water. Crude oil (10 µL) was added to form a thin layer on the surface and 10 µL of cell-free supernatant was applied on the oil surface. Uninoculated media and 10 µL (SDS 10%) were used as negative and positive control. The displaced diameter was measured, as described by Alvarez et al. [44].

#### 3.2.2. Drop-Collapsing Test

The drop-collapse test is a qualitative method to access biosurfactant production. Crude oil (2 µL) was dropped into each well of a 96-well micro plate and left overnight to reach equilibrium. Cell-free supernatant (5 µL) was dropped into the oil coated wells. Drop size with the magnifying glass was observed. The collapsed drop was recorded as positive for biosurfactant production and the beaded drop was an indicator that the sample does not produce biosurfactant as described by San et al. [45].

#### 3.2.3. Emulsification Index (E_24_) Measurement

Emulsification activity (E_24_) was measured as described by Chandankere et al. [18]. Different hydrocarbons and 72 h-old supernatant (5 mL each) were mixed in glass tubes and vortexed thoroughly for 2 minutes at high speed and maintained at room temperature for 24 h at static conditions. E_24_ was performed using hydrocarbons kerosene, hexadecane, tridecane, tetradecane, diesel, crude oil, pristine and heptane. Sodium Dodecyl Sulfate (0.1 g·L^−1^), Triton X (0.1% *v/v*) was used as control. The E_24_ was calculated by measuring the stable emulsion layer after 24 h at room temperature as follows:

Emulsification index E_24_ = (emulsified layer’s height/total height of the liquid) × 100

### 3.3. Identification of Selected Bacterial Isolate

For molecular identification, the isolated bacterial strain was sent to Beijing Liuhe Huada Gene Technology Co., Ltd. (Beijing, China) for sequencing using universal (16S rRNA) primers. The resulting sequence was compared with the sequence in the GeneBank database (NCBI) (https://blast.ncbi.nlm.nih.gov/Blast.cgi) and the phylogenetic tree was constructed through neighbor-joining methods using MEGA version 6.0 [46].

### 3.4. Media Optimization

The inoculum was prepared in LB medium for biosurfactant production studies, after 14 h incubation when the fermentation reached an optical density (OD660) of 0.8-0.9, 2% of inoculum was transferred to 250 mL flasks containing 50 mL of minimal media, and flasks were incubated in a shaker incubator (250 rpm at 37 °C). Samples were analyzed after 24 h for growth OD 600, pH, viscosity, ST and IFT. After 72 h, the previously-reported 7 different minimal media were analyzed for BS production, according to method of Al-Sulaimani et al. [3]. The composition of the different previously-reported media is tabulated in Table 5.

#### Carbon Source Optimization

Two selected media were further analyzed for the effect of carbon source on (growth OD600, pH, viscosity, surface tension, interfacial tension, Emulsification index (E_24_), and yield (g·L^−1^). Carbon sources (glucose, sucrose and starch soluble) were filtered separately before adding to the autoclaved media, 2% seed culture was added to 100 mL minimal media in 500 mL Erlenmeyer flasks, fermented in incubator shaker at 37 °C and 250 rpm. Samples were withdrawn at 24 h, 48 h and 72 h to analyze the growth, pH, viscosity, ST and IFT, yield and emulsification index (E24). Cell-free supernatant after centrifugation (1000× *g*) for 20 minutes was used for all measurements.

### 3.5. Determination of Surface Tension, Interfacial Tension and Alteration in Wettability

The surface tension of 24, 48 and 72-h-old cell-free supernatant was measured using Dataphysics (DCAT11, BW. Filderstadt, Germany) employing Du-Nouy’s ring method. The measurement was repeated thrice and each time the platinum ring was washed with distilled water and alcohol, then flamed until red. The interfacial tension of the cell-free supernatant against *n-heptane* was measured using the spinning drop interfacial tensiometer model (TX500C, CNG USA KINO Industry CO. Ltd.) at 25 °C and 6000 rpm and each result was average of triplicate. The contact angle was measured for both uninoculated media and cell-free supernatant using the Drop Shape Analysis System OCA20, (Dataphysics, Filderstadt, Germany) on hydrophobic surface cover slips at 25 °C and 1 atm pressure, according to the method of Al-Sulaimani et al. [3].

### 3.6. Biosurfactant Extraction and Partial Purification

Biosurfactant extraction was based on a combination of acid precipitation and solvent extraction as described by Joshi et al. [54]. Bacterial broth was centrifuged at 12,000× *g* for 20 min at 4 °C, collected cell-free supernatant was filtered through a (0.22 mm) pore-size filter and subjected to acid precipitation (HCl 6M). pH was adjusted to 2 and left at 4 °C for 12 h. Then, the overnight broth was centrifuged again (12,000× *g* for 20 min at 4 °C) and the collected pellet was washed twice with acidified water (pH2). The collected pellet was dried in a hot air oven at 65 °C and transferred to a 100 mL conical flask containing 50 mL methanol and left overnight with intermittent shaking, organic extracted was filtered thrice with methanol and was evaporated using rotary evaporator, a viscous matter was obtained and it was liquefied and pH adjusted to 8, then freeze-dried to obtain a light brown crude biosurfactant.

### 3.7. Critical Micelle Concentration (CMC) of Biosurfactant

Critical micelle concentration is the lowest amount of biosurfactant required to achieve maximum surface tension. Above this concentration, all biosurfactant tends to micelle formation and no further reduction in ST is achieved. For CMC determination, 1 g of freeze-dried biosurfactant was dissolved in Tris HCL-solution pH8 and a series of dilutions were prepared and surface tension was measured for each dilution, as described by Sharma et al. [55].

### 3.8. Effect of Environmental Factors on Production and Stability of Biosurfactant

In the first set of experiments, M7 media with glucose as carbon source were subjected to different internal and external environmental conditions like pH (2–12) using 6N-HCL/1 N HCL. Salinity (up to 0–30 g·L^−1^) of NaCl, temperature (20, 25, 30, 35, 37, 40, 45 and 50 °C), agitation speed (100, 150, 200, 250 and 300 rpm) and their effect on surface and interfacial tension (mN·m^−1^) respectively after 72 h of incubation were studied. Stability of 72 h old biosurfactant broth was measured by exposing broth to different temperatures (from 40–110 °C) overnight and at autoclave conditions (121 °C, 15Psi for 30 min), different NaCl concentrations (0–30 g·L^−1^) and pH (2.0–12.0). Surface tension and interfacial tension for pH and salinity was measured immediately and for various temperatures. ST and IFT were measured when the broth was stable at room temperature, according to the method of Hentati et al. [56].

### 3.9. Fourier Transform Infrared Spectroscopy (FT-IR)

For partial identification of structural groups, FTIR was used. The biosurfactant sample (1mg) was mixed with potassium bromide (KBR), pressed for 30 seconds and translucent pellets were obtained. Using a Perkin Elmer grating 100 IR (Norwalk, CT, USA) IR spectra were collected from 400-4000 wavenumbers (cm^−1^). 

### 3.10. Molecular Mass Determination of the Biosurfactant

MALDI-TOF mass spectrophotometry was carried out for mass-spectrophotometric analysis of the isolated biosurfactant, operating in the positive mode using UltaFlextreme (Bruker-Daltonics Bremen, Germany) in the *m/z* range of (50–2000 Da). Matrix preparation involves, mixing 2 µL of 2, 5 Dihydroxy benzoic acid (DHB) matrix 20 mL/mL in TA 30 (30:70 *v/v* (acetonitrile) ACN:TFA (triflouroacitic acid) 0.1% TFA) was premixed with 2 µL of the sample solution. One microliter of the sample solution was applied to the ground steel target plate and dried at room temperature. Flex software v3.3 (Bruker Daltonics, Germany) was used for visualization and initial data processing, as reported previously by Elshafie et al. [32].

### 3.11. Hydrocarbon (Motor Oil) Removal from Soil

Biosurfactant application for the removal of used motor oil from soil was tested as described previously by Hentati et al. [56]. Soil (50 g) contaminated with 5 grams (10% *v/w*) of used motor oil was transferred to 500 mL erlenmeyer flasks and were subjected to the following treatments: addition of 100 mL 72 h old supernatant using M7 media, 100 mL of Milli-Q water as control, 100 mL of solution of crude biosurfactant at CMC level (21 mg·L^−1^) and 100 mL of chemical surfactants at their CMC levels: Tween 80 = 0.0016 g, and Triton X-100 = 15.5 µL in 100 mL Milli-Q water respectively. The samples were incubated it at 30 °C and 150 rpm in a rotary shaker for 24 h. Samples were then centrifuged at 10,000× *g* for 20 min and the resulting supernatant was then extracted twice (*v/v*) with hexane, after the impact of surfactants the amount of the residue oil in the sand was gravimetrically calculated, the percentage of removed oil was calculated with Equation (1).

(1) $$Motor\;oil\;removed\;\left(\%\right)=\frac{Oi-Or}{Oi}\;\times 100$$

where *O_i_* is initial motor oil used in soil before washing (g), *O_r_* is the amount of oil left in soil after washing (g).

### 3.12. Enhanced Oil Recovery at Laboratory Scale

Sand pack column test for potential application of biosurfactant in EOR was constructed as described by Suthar et al. [40] with some modifications. Sand (300 g) of particle size 150 µm was filled with small amounts to ensure uniform packing in the glass column of inner diameter 45 × 50 × 175 mm. The column was obtained from (Bao Ru YI Biotechnology Co., Ltd. Beijing, China). Two sieves of 1mm pore size and 100 µm were placed in the upper and lower end, respectively, and tightly fixed at both ends with rubber caps with 1.2 mm holes for syringe insertion. Tight rubber rings were fixed around the caps for leakage prevention.

(i) Column saturation with brine: To ensure the removal of gases, nitrogen gas for 5 min was passed from the column and a vacuum was held for 5 min. Brine was then flooded at 7 kg·cm^−2^ air pressure, pore volume was calculated as the brine volume required for 100% saturation of the column. Three pore volume (PV) of brine was required for the column saturation.
(ii) Oil saturation of column: Light crude oil (Xinjiang crude oil, China) density 850 kg/m^3^ was passed through the sand pack column under pressure, as did earlier with brine until remaining brine saturation is reached. As oil passed in the column it displaced brine which was collected at the bottom end, Soi or initial saturation of oil was calculated by measuring the brine displaced by oil, also called OOIP (original oil in place).
(iii) Column flooding with brine: The column was flooded with brine again and the displaced oil was collected until there was no oil discharge from effluent. The PV of brine flooding was calculated to be about 6 to 9, termed as (Sor). The amount of oil retained was volumetrically determined by measuring the oil displaced. The residue oil saturation (Sor) was measured by assessing the displaced oil volume.
(iv) Biosurfactant flooding: Sand-packed columns were flooded with biosurfactant as described earlier for brine and oil, with 0.6 pore volumes of biosurfactant (CFS), flow rate was set to 3 mL/min. The biosurficant was passed through the columns, and the columns were incubated for 24 h following brine flooding. Column discharges were collected and the oil recovered using CFS flooding was measured. The oil recovery percentage was calculated using following formula,

PV (mL)=brine volume required for column saturation 

OOIP (mL)=brine volume displaced by oil saturation 

Sorwf (mL)=residual crude oil saturation after water flooding

Sorbf (%)=oil collected over residual oil saturation after biosurfactant flooding

(2) $$\mathrm{Swi}\;\left(\%\right)=\frac{x}{\mathrm{pv}}\times 100\left(x=\mathrm{pore}\;\mathrm{volume}-\mathrm{brine}\;\mathrm{collected}\;\mathrm{after}\;\mathrm{oil}\;\mathrm{injection}\right)$$

(3) $$\mathrm{Soi}\;\left(\%\right)=\frac{\mathrm{OOIP}}{\mathrm{PV}}\times 100$$

$$Sor\;\left(\%\right)=\frac{\mathrm{Xi}}{ooip}\times 100\;\;(\mathrm{Xi}=\mathrm{OOIP}-\mathrm{volume}\;\mathrm{of}\;\mathrm{oil}\;\mathrm{collected}\;\mathrm{after}\;\mathrm{water}\;\mathrm{flooding}$$

(4) $$\mathrm{AOR}\;\mathrm{over}\;\mathrm{Sorwf}\;\left(\%\right)=\;\frac{\mathrm{Sorbf}}{\mathrm{OOIP}-\mathrm{Sorwf}}\times 100$$

### 3.13. Statistical Analysis

All experiments were performed in triplicates, data are expressed as the mean ± Standard deviation (STDEV.S). A standard statistical software Origin version 8.0 (OriginLab, Massachusetts, USA) was used for all statistical analysis and graphs. 

## 4. Conclusions

The potent bacteria *B. licheniformis* Ali5 was isolated from soil. The isolated bacterial strain displayed the capability for biosurfactant production within 24 h in carbohydrate-based minimal media. The obtained biosurfactant was characterized as lipopeptide (lichenysin-A) and showed excellent surface and interfacial tension activity, and changed the wettability towards being more water-wet. It was found to be highly stable at extreme environmental conditions. The study also presents a comprehensive explanation about the construction and procedure of the sand-packed column, as a rapid, suitable and less-expensive technique to assess the MEOR potential of biosurfactant. It is found that the bacterial isolate recovered 32.10% entrapped crude oil from the sand-packed column. Therefore, the isolated bacteria and biosurfactant produced could be highly proficient for environmental applications like microbial enhanced oil recovery and hydrocarbon removal from a polluted environment. 

## Figures and Tables

**Table 1:** The growth (OD660), pH profile, biosurfactant production (ST = surface tension and IFT = interfacial tension) and Viscosity (mPa·s) profile of *B. licheniformis* Ali5 in carbohydrate based minimal production media.

Parameters	Time	Media 1	Media 2	Media 3	Media 4	Media 5	Media 6	Media 7
**Growth(OD_660_)**	0 h	0.07 ± 0.02	0.00 ± 0.0	0.02 ± 0.01	0.02 ± 0.01	0.20 ± 0.06	0.03 ± 0.04	0.06 ± 0.03
	24 h	1.42 ± 0.12	1.77 ± 0.04	0.77 ± 0.13	0.77 ± 0.13	1.45 ± 0.07	1.41 ± 0.08	1.64 ± 0.13
	48 h	1.56 ± 0.08	2.16 ± 0.07	0.85 ± 0.45	0.85 ± 0.45	1.52 ± 0.15	1.96 ± 0.21	2.15 ± 0.11
	72 h	1.53 ± 0.11	2.11 ± 0.03	0.94 ± 0.03	0.94 ± 0.03	1.48 ± 0.08	1.87 ± 0.08	2.11 ± 0.08
**pH**	0 h	7.25 ± 0.07	7.04 ± 0.04	7.08 ± 0.11	7.08 ± 0.11	7.04 ± 0.03	7.13 ± 0.09	7.28 ± 0.12
	24 h	7.18 ± 0.05	7.14 ± 0.04	5.95 ± 0.03	5.95 ± 0.03	6.64 ± 0.19	7.04 ± 0.24	8.06 ± 0.06
	48 h	7.05 ± 0.03	7.15 ± 0.10	5.75 ± 0.24	5.75 ± 0.24	6.63 ± 0.27	6.83 ± 0.19	8.86 ± 0.23
	72 h	7.05 ± 0.13	7.25 ± 0.03	4.80 ± 0.13	4.80 ± 0.13	6.71 ± 0.13	6.63 ± 0.20	9.46 ± 0.46
**ST mN·m^−1^**	0 h	71.13 ± 1.12	69.37 ± 0.37	68.92 ± 0.85	68.92 ± 0.85	69.34 ± 1.08	72.11 ± 0.65	73.06 ± 1.03
	24 h	48.48 ± 0.42	27.33 ± 0.51	40.64 ± 1.18	40.64 ± 1.18	36.45 ± 0.94	34.43 ± 0.61	26.37 ± 0.31
	48 h	37.44 ± 0.56	28.45 ± 0.71	50.67 ± 0.32	50.67 ± 0.32	31.70 ± 0.40	40.00 ± 0.84	26.63 ± 0.81
	72 h	35.57 ± 0.36	27.50 ± 0.29	57.48 ± 0.40	57.48 ± 0.40	31.04 ± 0.69	33.70 ± 1.17	26.55 ± 0.57
**IFT mN·m^−1^**	0 h	46.82 ± 0.23	39.52 ± 0.10	39.91 ± 0.35	39.91 ± 0.35	38.77 ± 0.37	46.53 ± 0.46	47.39 ± 0.11
	24 h	19.81 ± 0.18	2.60 ± 0.74	16.11 ± 0.23	16.11 ± 0.23	16.44 ± 0.28	9.6 ± 0.22	0.78 ± 0.13
	48 h	9.26 ± 0.28	3.72 ± 0.36	23.38 ± 0.69	23.38 ± 0.69	9.37 ± 0.24	14.72 ± 0.21	0.83 ± 0.16
	72 h	9.04 ± 0.52	3.49 ± 0.13	35.45 ± 0.20	35.45 ± 0.20	12.90 ± 0.16	10.63 ± 0.29	0.85 ± 0.20
**Viscosity**	0 h	1.14 ± 0.06	1.10 ± 0.03	1.19 ±0.07	1.19 ± 0.07	1.24 ± 0.04	1.09 ± 0.03	1.12 ± 0.05
	24 h	1.25 ± 0.03	1.26 ± 0.07	1.17 ± 0.06	1.17 ± 0.06	1.28 ± 0.05	1.38 ± 0.06	1.36 ± 0.04
	48 h	1.38 ± 0.04	1.36 ± 0.04	1.18 ± 0.08	1.18 ± 0.08	1.18 ± 0.08	1.44 ± 0.05	1.31 ± 0.07
	72 h	1.35 ± 0.07	1.29 ± 0.05	1.30 ± 0.05	1.30 ± 0.05	1.10 ± 0.06	1.32 ± 0.08	1.39 ± 0.03

All values are means, SD (*n* = 3). ST = surface tension, IFT = interfacial tension.

**Table 2:** The growth (OD660), pH profile, biosurfactant production (ST = surface tension and IFT = interfacial tension), viscosity (mPa·s), emulsification index (E_24_) and yield (g·L^−1^) profile of *B. licheniformis* Ali5 in M2 and M7 with different carbon sources.

Parameters	Time	M2 (Glucose)	M2 (Sucrose)	M2 (Starch)	M7 (Glucose)	M7 (Sucrose)	M7 (Starch)
**Growth (OD_660_)**	0 h	00 ± 0.00	0.05 ± 0.03	0.05 ± 0.05	0.30 ± 0.07	0.03 ± 0.02	0.07 ± 0.05
	24 h	1.71 ± 0.04	1.66 ± 0.05	1.33 ± 0.05	1.64 ± 0.05	1.84 ± 0.02	1.46 ± 0.13
	48 h	2.11 ± 0.02	1.81 ± 0.06	1.51 ± 0.04	2.19 ± 0.06	2.16 ± 0.04	1.91 ± 0.12
	72 h	2.08 ± 0.03	1.74 ± 0.02	1.61 ± 0.08	2.13 ± 0.01	2.11 ± 0.03	1.73 ± 0.05
**pH**	0 h	7.04 ± 0.04	7.09 ± 0.10	7.06 ± 0.07	7.28 ± 0.08	7.08 ± 0.36	7.11 ± 0.06
	24 h	7.12 ± 0.20	7.22 ± 0.06	7.28 ± 0.19	7.39 ± 0.13	7.22 ± 0.22	7.50 ± 0.24
	48 h	7.15 ± 0.17	7.28 ± 0.50	7.39 ± 0.25	8.49 ± 0.28	7.37 ± 0.04	8.48 ± 0.10
	72 h	7.43 ± 0.10	7.65 ± 0.10	7.33 ± 0.09	9.01 ± 0.12	8.76 ± 0.43	8.89 ± 0.28
**ST (** **mN·m^−1^)**	0 h	70.26 ± 0.29	71.63 ± 0.55	72.83 ± 0.36	72.24 ± 0.40	72.87± 0.48	71.72 ± 0.28
	24 h	27.66 ± 0.25	27.63 ± 0.35	29.60 ± 0.36	26.21 ± 0.33	26.49 ± 0.29	26.54 ± 0.34
	48 h	28.52 ± 0.55	27.70 ± 0.41	29.22 ± 0.29	26.48 ± 0.42	27.57 ± 0.25	27.36 ± 0.21
	72 h	27.57 ± 0.22	27.57 ± 0.14	29.40 ± 0.24	26.69 ± 0.17	26.55 ± 0.31	26.53 ± 0.14
**IFT (** **mN·m^−1^)**	0 h	47.86 ± 0.08	42.51 ± 0.09	47.19 ± 0.57	48.66 ± 0.18	48.48 ± 0.47	46.82 ± 1.39
	24 h	2.47 ± 0.18	1.41 ± 0.07	2.31 ± 0.23	0.26 ± 0.05	0.77 ± 0.02	0.56 ± 0.29
	48 h	4.61 ± 0.26	2.64 ± 0.14	2.71 ± 0.20	0.35 ± 0.07	0.82 ± 0.13	0.72 ± 0.14
	72 h	3.41 ± 0.14	2.10 ± 0.15	2.53 ± 0.29	0.37 ± 0.04	0.86 ± 0.03	0.78 ± 0.24
**Viscosity**	0 h	1.04 ± 0.05	1.12 ± 0.05	1.17 ± 0.09	1.14 ± 0.04	1.15 ± 0.02	1.18 ± 0.03
	24 h	1.44 ± 0.19	1.59 ± 0.09	1.24 ± 0.10	1.42 ± 0.07	1.32 ± 0.05	1.43 ± 0.07
	48 h	1.43 ± 0.09	1.46 ± 0.08	1.27 ± 0.04	1.35 ± 0.04	1.31 ± 0.04	1.31 ± 0.04
	72 h	1.33 ± 0.05	1.43 ± 0.08	1.25 ± 0.09	1.39 ± 0.06	1.31 ± 0.08	1.30 ± 0.05
**Emulsification index (E_24_)**		Diesel	Hexadecane	Heptane	Diesel	Hexadecane	Heptane
	0 h 24 h	19.8 ± 2.1 51.6 ± 1.0	10.6 ± 1.2 51.6 ± 0.6	12.7 ± 0.8 50.0 ± 0.9	18.3 ± 1.7 53.3 ± 0.7	12.8 ± 1.2 52.6 ± 0.3	10.4 ± 2.2 51.8 ± 0.6
	48 h	52.8 ± 0.7	52.5 ± 0.7	51.8 ± 1.1	53.3 ± 0.8	52.8 ± 0.2	52.2 ± 1.2
	72 h	53.0 ± 1.2	52.8 ± 0.5	52.7 ± 1.3	54.4 ± 0.9	53.8 ± 0.5	52.6 ± 0.8
**Yield (g·L^−1^)**	24 h	0.19 ± 0.2	0.22 ± 0.3	0.18 ± 0.3	0.29 ± 0.2	0.30 ± 0.4	0.36 ± 0.3
	48 h	0.45 ± 0.2	0.43 ± 0.2	0.48 ± 0.1	0.57 ± 0.3	0.66 ± 0.2	0.39 ± 0.2
	72 h	0.21 ± 0.1	0.48 ± 0.2	0.24 ± 0.2	1.01 ± 0.2	0.98 ± 0.1	0.55 ± 0.1

All values are means ± SD (*n* = 3). ST = surface tension, IFT = interfacial tension.

**Table 3:** Emulsification index (E_24_) of biosurfactant (cell-fee supernatant) produced from *B. licheniformis* Ali5, Triton X-100 and Sodium dodecyl sulfate against different hydrocarbons.

Hydrocarbon	Lichenysin	Triton X-100	SDS
kerosene	57.6 ± 0.9	51.8 ± 1.4	51.2 ± 0.6
hexadecane	53.8 ± 0.8	50.1 ± 0.2	50.6 ± 1.4
tridecane	55.4 ± 1.3	51.2 ± 1.6	52.3 ± 0.5
tetradecane	56.5 ± 0.2	48.8 ± 0.9	51.4 ± 1.6
diesel	54.5 ± 1.2	50.6 ± 0.5	53.8 ± 1.9
crude oil	66.4 ± 1.4	48.2 ± 1.0	45.2 ± 2.2
pristane	55.1 ± 1.4	49.8 ± 1.2	52.1 ± 1.3
heptane	52.4 ± 1.0	47.5 ± 1.3	52.4 ± 1.7

All values are means ± SD (*n* = 3). Sodium dodecyl sulfate (0.1 g/L). Triton X: concentration of 0.1% (*v/v*).

**Table 4:** Results obtained in enhanced oil recovery (EOR) sand-packed columns using biosurfactant (CFS) produced by *B. licheniformis* Ali5.

Sand-Packed Column Test								
Parameters	Biosurfactant Flooding				Control Flooding			
	SP 1	SP 2	SP 3	Mean ± SD	CT 1	CT 2	CT 3	Mean ± SD
PV (mL)	59	60	62	60.3 ± 1.52	58.0	59.0	61.0	59.3 ± 1.5
OOIP (mL)	46.4	45	48	46.5 ± 1.50	45.5	47.0	47.5	46.6 ± 1.0
S_oi_ (%)	78.6	75	77.4	77.0 ± 1.85	78.4	79.6	77.8	78.6 ± 0.9
S_wi_ (%)	21.4	25	22.5	23.0 ± 1.86	21.5	20.3	22.1	21.3 ± 0.9
S_orwf_ (mL)	23.6	24.3	26.4	24.8 ± 1.46	22.6	20.2	23.0	21.9 ± 1.5
OOIP-S_orwf_ mL	22.8	20.7	21.6	21.7 ± 1.05	22.9	26.7	24.5	24.7 ± 1.9
S_or_ (%)	49.1	46	45	46.7 ± 2.13	50.3	57.0	51.5	52.9 ± 3.6
S_orbf_ (mL)	7.5	7.9	8.5	8.0 ± 0.51	2.0	1.8	1.5	1.7 ± 0.3
AOR (%)	31.7	32.5	32.1	32.10 ± 0.4	8.7	6.7	6.1	7.1 ± 1.4

All values are means ± SD (*n* = 3). SP = sand pack column experiment. CFS = cell-free supernatant, CT = control (water) flooding, PV (mL) = brine volume required for column saturation, (OOIP, mL) = original oil in place, S_oi_ = initial oil saturation, S_wi_ = water required for column saturation, S_orwf_ (mL) = oil recovered after water flooding, S_or_ = residual oil saturation, S_orbf_ (%) = oil collected over residual oil saturation after biosurfactant flooding, AOR = additional oil recovered.

**Table 5:** The composition of different previously reported minimal media.

Reference	[47]	[48]	[49]	[50]	[51]	[52]	[53]
Composition g/L	Media 1	Media 2	Media 3	Media 4	Media 5	Media 6	Media 7
**Glucose**	34.0	11.0	20.0	20.0	10.0	20.0	-
**Sucrose**	-	-	-	-	-	-	20.0
**KH_2_PO_4_**	6.0	-	1.0	-	-	4.8	0.14
**K_2_HPO_4_**	-	-	-	-	2.7	-	2.2
**Na_2_HPO_4_**	1.0	-	-	-	-	7.12	-
**NH_4_NO_3_**	1.0	-	-	-	-	4.0	3.3
**NaNO_3_**	-	4.4	-	2.8	-	-	-
**MgSO_4_·7H_2_O**	0.1	0.8	0.5	0.20	0.25	0.2	0.6
**FeSO_4_·7H_2_O**	0.0016	-	-	-	-	0.0011	0.1
**MnSO_4_·4H_2_O**	0.0012	-	0.005	-	-	0.0006	-
**CaCl_2_**	0.0012	-	-	-	-	0.0007	0.01
**EDTA**	0.0007	-	-	0.2	-	0.0014	-
**KCl**	-	0.4	-	-	-	-	-
**H3PO4 (85.4%)**	-	1.0 mL/L	-	0.5 mL/L	-	-	-
**C_5_H_8_NO_4_Na**	-	-	5.0	-	-	-	-
**CuSO_4_**	-	-	0.2	-	-	-	-
**(NH_4_)_2_SO_4_**	-	-	-	-	1.0	-	-
**Yeast extract**	-	-	1.0	-	1.0	-	-
**NaCl**	-	-	-	-	5.0	-	0.01
**Trace elements**	-	10.0 mL/L	-	1.0 mL/L	-	-	0.5 mL/L
